# Correction: Similarity and dissimilarity in alterations of the gene expression profile associated with inhalational anesthesia between sevoflurane and desflurane

**DOI:** 10.1371/journal.pone.0339699

**Published:** 2025-12-23

**Authors:** Takehiro Nogi, Kousuke Uranishi, Ayumu Suzuki, Masataka Hirasaki, Tina Nakamura, Tomiei Kazama, Hiroshi Nagasaka, Akihiko Okuda, Tsutomu Mieda

In the Animal experiments subsection of the Materials and methods, there is an error in the last sentence of the paragraph. The correct sentence is: The protocol for these experiments was approved by the Institutional Animal Care and Use Committee of Saitama Medical University (permission numbers 3301, 3547 and 3796).
