# Supplementary figures and images for: Similarity and dissimilarity in alterations of the gene expression profile associated with inhalational anesthesia between sevoflurane and desflurane
Source: PLoS One. 2024 Mar 28;19(3):e0298264. doi: 10.1371/journal.pone.0298264 (PMC10977671; doi:10.1371/journal.pone.0298264)

**A**

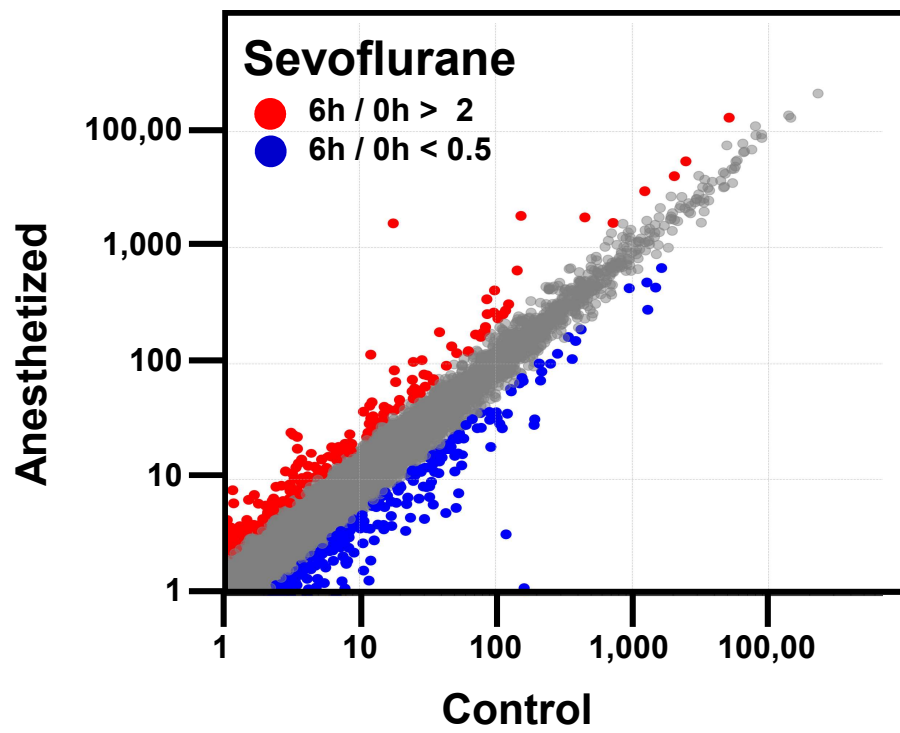

**B**

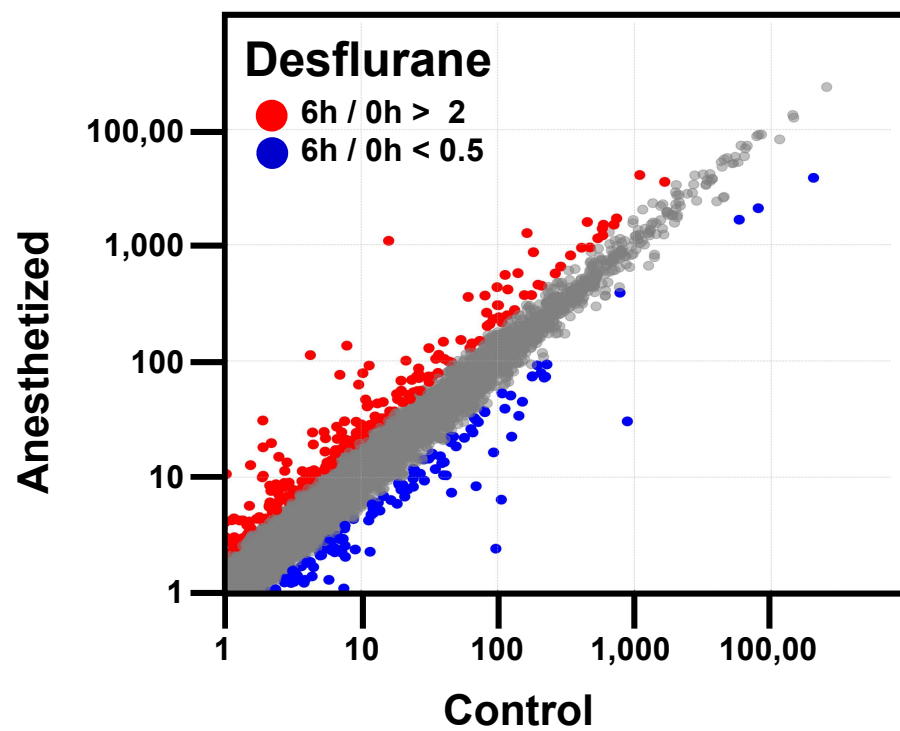

Supplement: S1 Fig — Upper and lower panels show data obtained from experiments of inhalational anesthesia using sevoflurane and desflurane, respectively. These scatter plots were generated after removing genes whose lengths are equal or shorter than 200 base pairs from the gene list of RNA sequence data. Numerical values shown on the X- and Y-axes are TPM values from RNA sequence data. Genes whose TPM values were increased or decreased more than 2-fold by inhalational anesthesia using sevoflurane or desflurane are indicated as red and blue dots, respectively. The numbers of genes upregulated by 6 hours treatment with sevoflurane and desflurane were 210 and 282, respectively, of which 59 genes overlapped, and 329 and 141 genes were downregulated by sevoflurane and desflurane treatments, respectively, with 31 overlapping genes. S1 Table shows a list of these genes with their official gene symbols. (PDF) [file pone.0298264.s001.pdf]

**A**

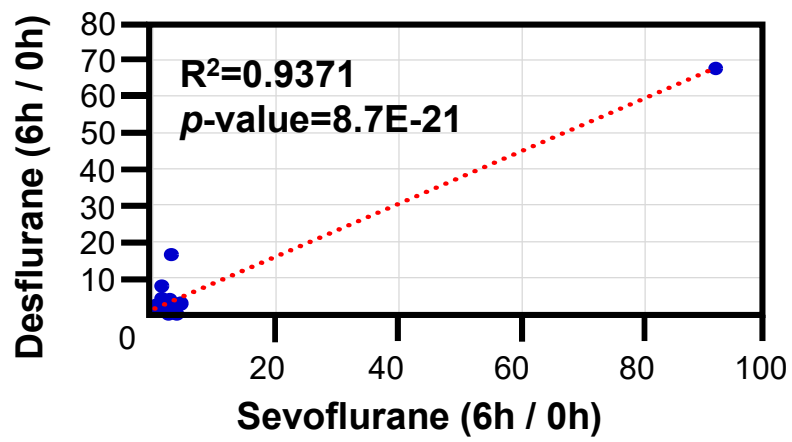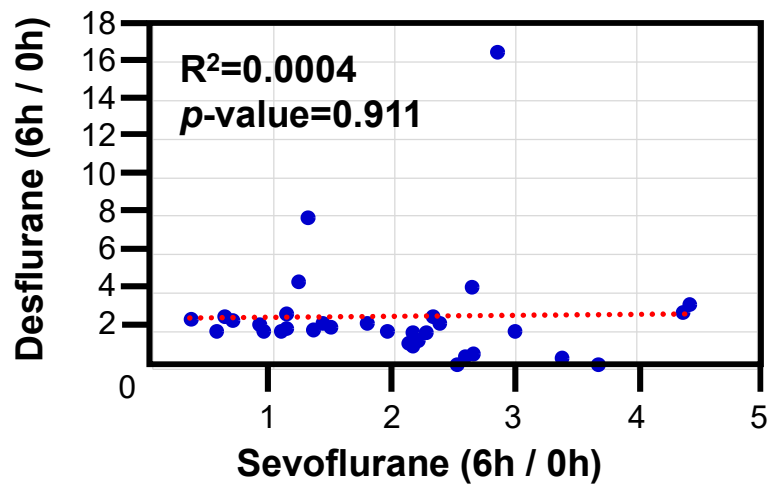

**B**

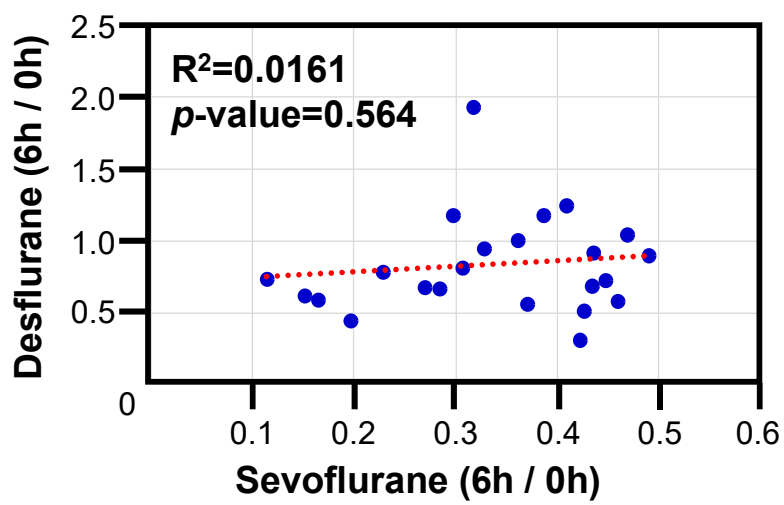

Supplement: S2 Fig — (A, B) Coefficient of determination was calculated using the genes in Fig 1D that were upregulated by sevoflurane and/or desflurane more than 2-fold among the members of the GO term “response to xenobiotic stimulus (0009410)” (A, upper panel) and genes shown in Fig 2D that were downregulated by sevoflurane more than 2-fold among the members of the GO term “defense response to virus (0051607)” (B). Lower panel in A shows the result after the removal of Cyp2b1 gene data as an outlier in the gene set. (PDF) [file pone.0298264.s002.pdf]

A

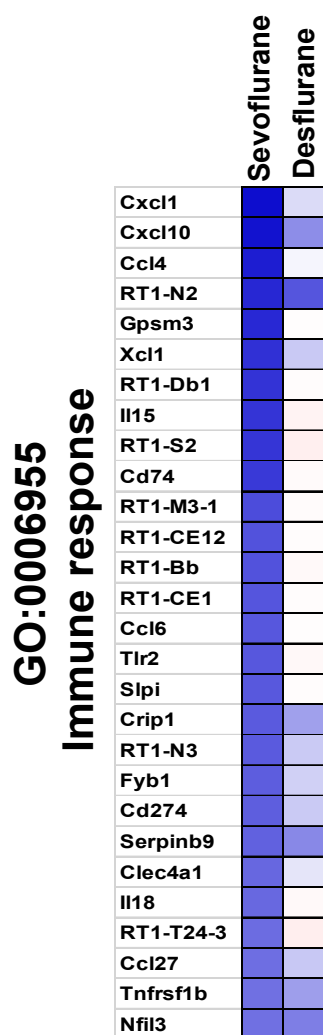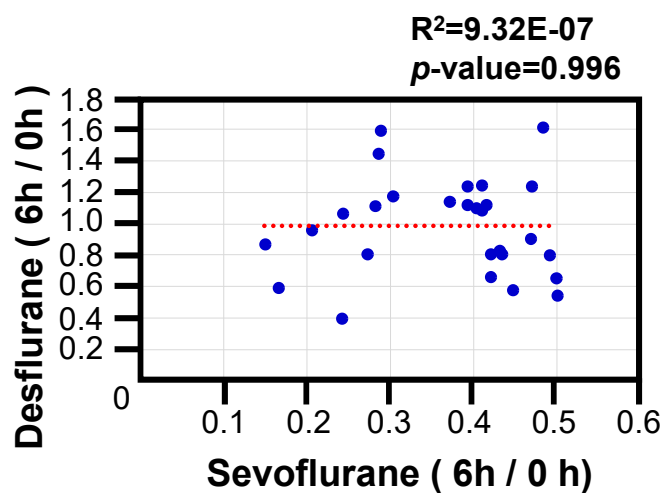

B

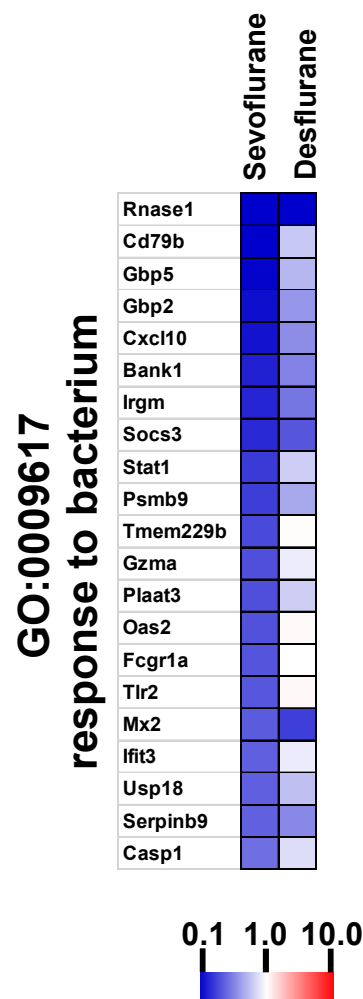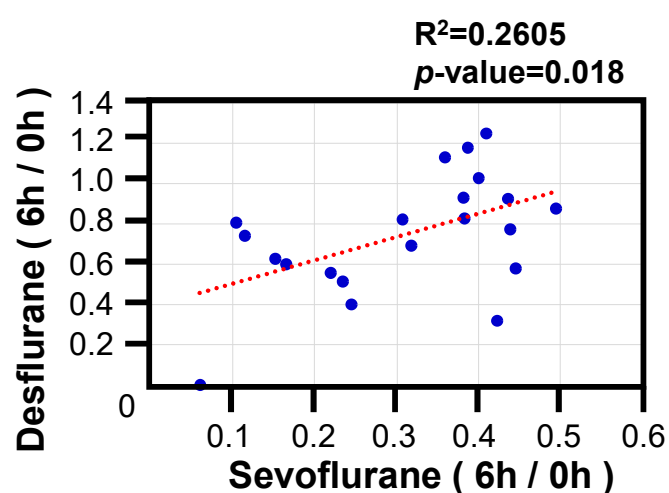

Supplement: S3 Fig — (A, B) Effect of desflurane treatment on the expressions of genes that contributed to the identification of immune response-related terms as sevoflurane treatment-specific GO terms. Genes downregulated more than 2-fold by sevoflurane treatment were selected among genes constituting the GO terms “immune response (0006952)” (A) and “response to bacterium (0009617)” (B). Relative expression levels in the livers of rats treated with desflurane for 6 hours compared to the control were demonstrated by a heatmap (right column) along with data obtained by the analyses of livers of rats treated with sevoflurane (left column). Panels shown under each heatmap represent regression analyses for the calculation of the coefficient of determination. (PDF) [file pone.0298264.s003.pdf]
