# Supplementary material for: Similarity and dissimilarity in alterations of the gene expression profile associated with inhalational anesthesia between sevoflurane and desflurane
Source: PLoS One. 2024 Mar 28;19(3):e0298264. doi: 10.1371/journal.pone.0298264 (PMC10977671; doi:10.1371/journal.pone.0298264)
Supplement: S4 Fig — (A) A list of gene sets identified by GSEA as significantly activated gene sets by inhalational anesthesia using sevoflurane or desflurane. Three distinct publicly available databases, “biological process of Gene Ontology”, “Kyoto Encyclopedia of Genes and Genome”, and “Reactome Pathway Database” were used for the analyses, in which the top twenty terms according to their NES values were selected from positively-regulated gene sets with the analyses using each platform, but terms whose p-values were greater than 0.05 were eliminated from the list. Terms related to xenobiotic metabolism are shown in light blue. (B) A list of gene sets identified by GSEA as significantly repressed gene sets by inhalational anesthesia using sevoflurane or desflurane. The same criteria used in (A) were used. Commonly identified gene sets by treatment with sevoflurane or desflurane are shown in green. Terms related to immune response are indicated by red font. (PDF) [file pone.0298264.s004.pdf]

A

GO-BP

Activated by sevoflurane	NES	*p*-value	Activated by desflurane	NES	*p*-value
RIBOSOME_BIOGENESIS	2.40	0	SYNAPTIC_TRANSMISSION_CHOLINERGIC	2.31	0
PRESYNAPSE_ORGANIZATION	2.36	0.002	CYCLIC_NUCLEOTIDE_BIOSYNTHETIC_PROCESS	2.24	0
POSITIVE_REGULATION_OF_DENDRITIC_SPINE_DEVELOPMENT	2.29	0	CYCLIC_NUCLEOTIDE_METABOLIC_PROCESS	2.2	0.001
REGULATION_OF_DENDRITIC_SPINE_DEVELOPMENT	2.25	0	LIGAND_GATED_ION_CHANNEL_SIGNALING_PATHWAY	2.15	0.001
POSITIVE_REGULATION_OF_EXCITATORY_POSTSYNAPTIC_POTENTIAL	2.24	0	CGMP_METABOLIC_PROCESS	2.09	0.003
SYNAPSE_MATURATION	2.24	0	IONOTROPIC_GLUTAMATE_RECEPTOR_SIGNALING_PATHWAY	2.06	0.004
RRNA_METABOLIC_PROCESS	2.22	0	HOMOPHILIC_CELL_ADHESION_VIA_PLASMA_MEMBRANE_ADHESION_MOLECULES	2.06	0
INTERMEDIATE_FILAMENT_BASED_PROCESS	2.19	0	BIOLOGICAL_PROCESS_INVOLVED_IN_INTRASPECIES_INTERACTION_BETWEEN_ORGANISMS	2.04	0.004
MAINTENANCE_OF_SYNAPSE_STRUCTURE	2.19	0	SENSORY_PERCEPTION_OF_SMELL	2.04	0
MATURATION_OF_SSU_RRNA	2.18	0.002	SEROTONIN_RECEPTOR_SIGNALING_PATHWAY	2.03	0.008
RIBOSOMAL_SMALL_SUBUNIT_BIOGENESIS	2.17	0	CGMP_MEDIATED_SIGNALING	2.01	0.007
LONG_CHAIN_FATTY_ACID_TRANSPORT	2.16	0.002	STABILIZATION_OF_MEMBRANE_POTENTIAL	2.01	0.006
METAPHASE_CHROMOSOME_ALIGNMENT	2.16	0	MEMBRANE_PROTEIN_PROTEOLYSIS	2	0.001
POSTSYNAPTIC_MEMBRANE_ORGANIZATION	2.15	0	URETER_DEVELOPMENT	1.99	0.007
KERATINIZATION	2.15	0.005	G_PROTEIN_COUPLED_SEROTONIN_RECEPTOR_SIGNALING_PATHWAY	1.98	0.01
RIBOSOMAL_LARGE_SUBUNIT_BIOGENESIS	2.14	0.002	OOCYTE_MATURATION	1.97	0.012
POSITIVE_REGULATION_OF_SYNAPTIC_TRANSMISSION_GLUTAMATERGIC	2.13	0.007	MEMBRANE_PROTEIN_ECTODOMAIN_PROTEOLYSIS	1.94	0.011
REGULATION_OF_SYNAPSE_ASSEMBLY	2.12	0	BLOOD_VESSEL_REMODELING	1.94	0.011
POSITIVE_REGULATION_OF_SYNAPTIC_TRANSMISSION_GABAERGIC	2.12	0	REGULATION_OF_CELL_MATURATION	1.94	0.017
DENDRITIC_SPINE_DEVELOPMENT	2.09	0.003	GLUCOCORTICOID_BIOSYNTHETIC_PROCESS	1.92	0.007

KEGG

Activated by sevoflurane	NES	*p*-value	Activated by desflurane	NES	*p*-value
OXIDATIVE_PHOSPHORYLATION	1.99	0.003	OLFACTORY_TRANSDUCTION	2.14	0
SPLICEOSOME	1.71	0.015	DRUG_METABOLISM_CYTOCHROME_P450	1.74	0.037
AMINOACYL_TRNA_BIOSYNTHESIS	1.69	0.037	TRYPTOPHAN_METABOLISM	1.71	0.039
OOCYTE_MEIOSIS	1.55	0.043	LINOLEIC_ACID_METABOLISM	1.67	0.040
CELL_CYCLE	1.51	0.038	STEROID_HORMONE_BIOSYNTHESIS	1.66	0.042
			NEUROACTIVE_LIGAND_RECEPTOR_INTERACTION	1.64	0.004
			DRUG_METABOLISM_OTHER_ENZYMES	1.62	0.042

REACTOME

Activated by sevoflurane	NES	*p*-value	Activated by desflurane	NES	*p*-value
RKERATINIZATION	2.23	0.002	OLFACTORY_SIGNALING_PATHWAY	2.18	0
RRNA_MODIFICATION_IN_THE_NUCLEUS_AND_CYTOSOL	2.19	0	FERTILIZATION	1.96	0.011
FORMATION_OF_THE_CORNIFIED_ENVELOPE	2.15	0	PHASE_4_RESTING_MEMBRANE_POTENTIAL	1.90	0.008
HSF1_DEPENDENT_TRANSACTIVATION	2.09	0.007	XENOBIOTICS	1.90	0.012
IRON_UPTAKE_AND_TRANSPORT	2.03	0.005	CARDIAC_CONDUCTION	1.88	0.009
CYTOSOLIC_TRNA_AMINOACYLATION	2.03	0.008	AMINE_LIGAND_BINDING_RECEPTORS	1.87	0.014
METABOLISM_OF_AMINE_DERIVED_HORMONES	2.02	0.005	THE_PHOTOTRANSDUCTION_CASCADE	1.85	0.022
ATTENUATION_PHASE	1.98	0.008	NA_CL_DEPENDENT_NEUROTRANSMITTER_TRANSPORTERS	1.84	0.018
RRNA_PROCESSING	1.95	0.001	ENDOGENOUS_STEROLS	1.74	0.036
TRANSFERRIN_ENDOCYTOSIS_AND_RECYCLING	1.95	0.018	MUSCLE_CONTRACTION	1.74	0.003
MITOTIC_SPINDLE_CHECKPOINT	1.93	0	NETRIN_1_SIGNALING	1.74	0.034
RESOLUTION_OF_SISTER_CHROMATID_COHESION	1.93	0.005	GLUCONEOGENESIS	1.72	0.045
ACETYLCHOLINE_NEUROTRANSMITTER_RELEASE_CYCLE	1.93	0.003	SENSORY_PERCEPTION	1.70	0
CELLULAR_RESPONSE_TO_HEAT_STRESS	1.93	0.005	METABOLISM_OF_AMINE_DERIVED_HORMONES	1.68	0.042
RESPONSE_OF_EIF2AK1_HRI_TO_HEME_DEFICIENCY	1.89	0.01	PHASE_I_FUNCTIONALIZATION_OF_COMPOUNDS	1.60	0.049
REGULATION_OF_HSF1_MEDIATED_HEAT_SHOCK_RESPONSE	1.86	0.008	VISUAL_PHOTOTRANSDUCTION	1.58	0.038
MITOTIC_PROMETAPHASE	1.83	0.003			
TRNA_AMINOACYLATION	1.81	0.022			
UPTAKE_AND_ACTIONS_OF_BACTERIAL_TOXINS	1.81	0.018			
HSF1_ACTIVATION	1.8	0.016			

B

GO-BP

Repressed by sevoflurane	NES	*p*-value	Repressed by desflurane	NES	*p*-value
ADAPTIVE_IMMUNE_RESPONSE	-2.60	0	DEFENSE_RESPONSE_TO_BACTERIUM	-2.09	0
DETECTION_OF_STIMULUS_INVOLVED_IN_SENSORY_PERCEPTION	-2.57	0	REFLEX	-2.08	0
LYMPHOCYTE_MEDIATED_IMMUNITY	-2.36	0	FEAR_RESPONSE	-2.06	0
LEUKOCYTE_MEDIATED_IMMUNITY	-2.35	0	GROOMING_BEHAVIOR	-2.06	0
REGULATION_OF_ADAPTIVE_IMMUNE_RESPONSE	-2.32	0	KERATINIZATION	-2.05	0
NEUTROPHIL_CHEMOTAXIS	-2.32	0	ANTIMICROBIAL_HUMORAL_IMMUNE_RESPONSE_MEDIATED_BY_ANTIMICROBIAL_PEPTIDE	-1.99	0
MYELOID_LEUKOCYTE_ACTIVATION	-2.30	0	L_GLUTAMATE_IMPORT	-1.95	0.004
ADAPTIVE_IMMUNE_RESPONSE_BASED_ON_SOMATIC_RECOMBINATION_OF_IMMUNE_RECEPTORS_BUILT_FROM_IMMUNOGLOBULIN_SUPERFAMILY_DOMAINS	-2.30	0	ADAPTATION_OF_SIGNALING_PATHWAY	-1.95	0
IMMUNE_RESPONSE_REGULATING_CELL_SURFACE_RECEPTOR_SIGNALING_PATHWAY	-2.29	0	NEGATIVE_ADAPTATION_OF_SIGNALING_PATHWAY	-1.94	0
TYPE_2_IMMUNE_RESPONSE	-2.28	0	STARTLE_RESPONSE	-1.92	0.003
REGULATION_OF_LEUKOCYTE_MEDIATED_IMMUNITY	-2.28	0	POSITIVE_REGULATION_OF_CALCIUM_ION_TRANSPORT	-1.90	0
NEUTROPHIL_MIGRATION	-2.27	0	NEGATIVE_REGULATION_OF_ENDOTHELIAL_CELL_PROLIFERATION	-1.90	0.003
GRANULOCYTE_MIGRATION	-2.26	0	RESPONSE_TO_CHEMOKINE	-1.89	0
ALPHA_BETA_T_CELL_ACTIVATION	-2.25	0	NEURONAL_ACTION_POTENTIAL	-1.87	0.007
GRANULOCYTE_CHEMOTAXIS	-2.25	0	L_GLUTAMATE_TRANSMEMBRANE_TRANSPORT	-1.87	0
REGULATION_OF_LYMPHOCYTE_MEDIATED_IMMUNITY	-2.21	0	THYROID_GLAND_DEVELOPMENT	-1.87	0
T_CELL_ACTIVATION_INVOLVED_IN_IMMUNE_RESPONSE	-2.19	0	CAMERA_TYPE_EYE_MORPHOGENESIS	-1.86	0
REGULATION_OF_B_CELL_ACTIVATION	-2.16	0	MACROPHAGE_ACTIVATION	-1.85	0
POSITIVE_REGULATION_OF_LEUKOCYTE_MEDIATED_IMMUNITY	-2.16	0	POSITIVE_REGULATION_OF_BLOOD_CIRCULATION	-1.85	0.003
T_CELL_SELECTION	-2.16	0	NEURAL_RETINA_DEVELOPMENT	-1.85	0.004

KEGG

Repressed by sevoflurane	NES	*p*-value	Repressed by desflurane	NES	*p*-value
CYTOKINE_CYTOKINE_RECEPTOR_INTERACTION	-2.37	0	DNA_REPLICATION	-1.75	0.006
HEMATOPOIETIC_CELL_LINEAGE	-2.18	0	P53_SIGNALING_PATHWAY	-1.66	0.013
INTESTINAL_IMMUNE_NETWORK_FOR_IGA_PRODUCTION	-2.04	0	CHEMOKINE_SIGNALING_PATHWAY	-1.51	0.008
ASTHMA	-2.04	0	TYPE_II_DIABETES_MELLITUS	-1.49	0.047
JAK_STAT_SIGNALING_PATHWAY	-2.01	0	SYSTEMIC_LUPUS_ERYTHEMATOSUS	-1.44	0.070
ALLOGRAFT_REJECTION	-2.00	0	T_CELL_RECEPTOR_SIGNALING_PATHWAY	-1.44	0.043
GRAFT_VERSUS_HOST_DISEASE	-1.96	0.003	CYTOKINE_CYTOKINE_RECEPTOR_INTERACTION	-1.36	0.018
AUTOIMMUNE_THYROID_DISEASE	-1.95	0.003			
LEISHMANIA_INFECTION	-1.92	0.005			
PRIMARY_IMMUNODEFICIENCY	-1.92	0.005			
SYSTEMIC_LUPUS_ERYTHEMATOSUS	-1.91	0.003			
FC_GAMMA_R_MEDIATED_PHAGOCYTOSIS	-1.90	0			
NATURAL_KILLER_CELL_MEDIATED_CYTOTOXICITY	-1.86	0			
CHEMOKINE_SIGNALING_PATHWAY	-1.84	0			
ANTIGEN_PROCESSING_AND_PRESENTATION	-1.83	0.005			
TYPE_II_DIABETES_MELLITUS	-1.72	0.013			
NOD_LIKE_RECEPTOR_SIGNALING_PATHWAY	-1.67	0.014			
OLFACTORY_TRANSDUCTION	-1.66	0			
TYPE_I_DIABETES_MELLITUS	-1.66	0.031			
AMYOTROPHIC_LATERAL_SCLEROSIS_ALS	-1.65	0.022			

REACTOME

Repressed by sevoflurane	NES	*p*-value	Repressed by desflurane	NES	*p*-value
OLFACTORY_SIGNALING_PATHWAY	-2.53	0	ANTIMICROBIAL_PEPTIDES	-2.54	0
INTERFERON_ALPHA_BETA_SIGNALING	-2.21	0	CHEMOKINE_RECEPTORS_BIND_CHEMOKINES	-1.95	0
IMMUNOREGULATORY_INTERACTIONS_BETWEEN_A_LYMPHOID_AND_A_NON_LYMPHOID_CELL	-2.21	0	PIWI_INTERACTING_RNA_PIRNA_BIOGENESIS	-1.93	0
CHEMOKINE_RECEPTORS_BIND_CHEMOKINES	-2.09	0	PEPTIDE_LIGAND_BINDING_RECEPTORS	-1.82	0
GENERATION_OF_SECOND_MESSENGER_MOLECULES	-2.07	0	CHAPERONE_MEDIATED_AUTOPHAGY	-1.79	0.003
INTERLEUKIN_10_SIGNALING	-1.99	0.002	RHO_GTPASES_ACTIVATE_FORMINS	-1.78	0.006
TNFS_BIND_THEIR_PHYSIOLOGICAL_RECEPTORS	-1.99	0	RESOLUTION_OF_SISTER_CHROMATID_COHESION	-1.77	0
PD_1_SIGNALING	-1.99	0	INTERLEUKIN_10_SIGNALING	-1.77	0.003
STRIATED_MUSCLE_CONTRACTION	-1.97	0	DEFENSINS	-1.76	0.006
CLASS_A_1_RHODOPSIN_LIKE_RECEPTORS	-1.94	0	RHO_GTPASE_EFFECTORS	-1.75	0
INTERFERON_GAMMA_SIGNALING	-1.92	0.003	BETA_DEFENSINS	-1.72	0.007
INTERLEUKIN_2_FAMILY_SIGNALING	-1.82	0.011	MITOTIC_PROMETAPHASE	-1.72	0
COSTIMULATION_BY_THE_CD28_FAMILY	-1.76	0.005	DNA_STRAND_ELONGATION	-1.71	0.015
TCR_SIGNALING	-1.75	0.003	INTERLEUKIN_12_FAMILY_SIGNALING	-1.70	0.010
TNFR2_NON_CANONICAL_NF_KB_PATHWAY	-1.75	0.014	REGULATION_OF_PLK1_ACTIVITY_AT_G2_M_TRANSITION	-1.62	0.022
GPCR_LIGAND_BINDING	-1.75	0	AURKA_ACTIVATION_BY_TPX2	-1.62	0.004
THE_ROLE_OF_NEF_IN_HIV_1_REPLICATION_AND_DISEASE_PATHOGENESIS	-1.73	0.018	MITOTIC_SPINDLE_CHECKPOINT	-1.59	0.02
COMPLEMENT_CASCADE	-1.72	0.02	INTERLEUKIN_12_SIGNALING	-1.59	0.007
DAP12_INTERACTIONS	-1.71	0.01	FORMATION_OF_THE_BETA_CATENIN_TCF_TRANSACTIVATING_COMPLEX	-1.56	0.014
SIALIC_ACID_METABOLISM	-1.71	0.011	ACTIVATION_OF_BH3_ONLY_PROTEINS	-1.56	0.034
