# Supplementary material for: Similarity and dissimilarity in alterations of the gene expression profile associated with inhalational anesthesia between sevoflurane and desflurane
Source: PLoS One. 2024 Mar 28;19(3):e0298264. doi: 10.1371/journal.pone.0298264 (PMC10977671; doi:10.1371/journal.pone.0298264)
Supplement: S1 Table — (PDF) [file pone.0298264.s005.pdf]

Table S1

Gene lists that were up- or down-regulated by sevoflurane and/or desflurane

Up-regulated			Down-regulated		
Group 1*	Group 2	Group 3	Group 4	Group 5	Group 6
(59 genes)	(151 genes)	(223genes)	(31 genes)	(298 genes)	(110 genes)
Ptgis	Ptprn	Colq	Gadd45g	Amy2a3	Gck
Cyp2b1	Prss32	Upp2	Hamp	Adh6	Ptprn
Gstm3l	Cux2	Clca5	Cish	Cel	Wfdc21
Cyp2b2	Slc41a3	Ebf4	Pnpla3	RGD1560242	Akr1b8
Gadd45b	Abcd2	Adcy10	Mcpt8l2	Cpa1	Rprm
RGD1311946	Fgf21	Zmynd12	Lyc2	Cela2a	Rp1
Nptx2	Wfdc21	LOC103692984	Irs3	Mmd2	Pde4d
Kcnn1	Slc7a11	Tex36	Ihh	Cpa2	Gimd1
Trim36	Igfbp6	Cyp2c24	Tsc22d1	Cpb1	Krt2
Optc	Rbp7	Rgs16	Mx2	Rnase1	Slc34a2
Mfsd2a	Noct	Egr1	Inhba	Pcdhac2	Esd
Got1	LOC108348108	RGD1359290	Osgin1	Arhgef37	Dusp1
Por	Akr1b8	Hcst	Bmf	Cd79b	Bmerb1
Alas1	Mthfd2	Cdh17	Stac3	Cdh17	Rn18s
Fam180a	Pde4d	Slc2a12	Tubb2b	MGC108823	Srms
Cyp3a23/3a1	Cox6b2	Eif4ebp3	Unc5cl	Chodl	Plac9
Trib3	Nhlrc1	Aldh3b1	RT1-N1	Gbp5	Csmd1
Zbtb16	Hspa1b	Snrpg	RT1-N2	Tnfrsf14	Mybl1
Ces2c	Tsku	Hacl1	Socs3	RGD1562890	Ctsw
Zfp354a	Slc25a25	Per1	Nrep	RGD1309808	Ppp1r3b
Sds	Slc7a1	Inmt	Gclm	Cfap126	Rn45s
Ankrd33b	Adm2	Fmo2	Slc13a4	Pcdha1	Tubb2a
Ttc25	Asns	Fbxo27	Dnajc12	Pcdha2	Rasd1
Scnn1a	Mllt11	G6pc	RGD1309362	Kantr	Rn28s
St8sia1	Lcn2	Slc25a47	Arntl2	Pcdha8	H2ac1
Unc5b	Phgdh	Ppargc1a	Edar	LOC100909539	Mex3b
Edem1	Coro6	Rdh16	Irf1	Pcdha12	Cd300c2
Gpd1l	Psat1	LOC102547290	MGC105567	Pcdha6	Lcn2
Cyp17a1	Ssx2ip	Meox2	Cml2	Pcdhac1	Mcm6
Tmem30b	Pdxp	Cyp3a18	Efna4	Cxcl1	Cyp4a2
Igfbp2	Ccnb1	Vnn1	Pparg	Pcdha11	Ltc4s
Rhbdd2	Ppic	Slc22a5		Gbp2	Fabp5
Pla2g12a	Tpm2	Gfra1		Hk3	Rtn4rl1
Chmp4c	Arl4d	RGD1563263		Ppp2r2b	Aldh1b1
Ptp4a1	Polg2	LOC106182250		Pcdha7	Angptl8
LOC684871	Ddit4	Gpat3		Pcdha3	Sebox
Plk3	G0s2	Trpm4		Adh4	Abo3
Cpt1a	Clec2dl1	Cyp3a9		Pcdha9	Fkbp4
RGD1563888	Terc	Mt2A		Pcdha13	Tuba4a
Slc25a22	Gpcpd1	Olr35		Cxcl10	Casp12
Nceh1	Wdpcp	Rab30		LOC100911796	Isg15
Bhmt	Chac1	Pdlim2		Pcdha5	Rcan1
Fmo3	Polr1e	Cpb1		Shisa3	Hes6
Ifrd1	Sert1	Klf13		Adora3	Otub2
RGD1562310	Inhbe	Clcf1		Ly6c	Hist1h2bd
Asl	Gnat1	Cib2		Olr1585	Chac1

* Group 1: Genes activated commonly by sevoflurane and desflurane
Group 2: Genes activated specifically by sevoflurane
Group 3: Genes activated specifically by desflurane
Group 4: Genes repressed commonly by sevoflurane and desflurane
Group 5: Genes repressed specifically by sevoflurane
Group 6: Genes repressed specifically by desflurane

Trmt61a	Knstrn	Pnpla2
Abcb1a	Mlc1	Ackr2
Ptdss2	Cenps	Cidea
Zfp36	Asrgl1	Sost
Mcm10	Mbnl3	Ech1
Ablim3	Spag8	Bhlha15
Dus4l	Ppl	Gys2
Retreg1	Sarm1	Usp2
Arl11	Cby1	Gcnt2
Igfbp1	Abcc3	Kcnk3
LOC100911627	Vldlr	Gde1
Slc2a5	Morn2	Depp1
Pnpla7	Wsb1	Pfkfb1
	Pclaf	Lpin1
	Fabp3	Hykk
	Rn18s	Lenep
	Them4	Nr1i2
	Wdr77	Abcg5
	Dip2a	Slc25a32
	Coq10b	Smox
	Paqr3	Ppcs
	Cyp4a2	Ccdc77
	Wfdc3	Entpd2
	Acta2	Abcg8
	Abo3	Angptl4
	RGD1560010	Zfp580
	Rgs10	LOC687707
	Actg1	Nemp1
	LOC498154	Ca5a
	Card19	Ptn
	Palm2	Slc35g1
	Dlat	Selp
	Poc1a	Tfap4
	Mid1	Shq1
	Cldn20	Dnase2b
	Thg1l	Pbsn
	Plin5	Tspan4
	Aco2	Omd
	Hand2	Phyh
	Ccnb2	Aptr
	Fitm2	Gpatch4
	Aass	Castor1
	Dancr	Pptc7
	Tgfb3	Tas1r1
	Tagln	Sulf2
	Ckap2	Nfkbiz
	Fam222a	Lipe
	Rpp38	Sult2a2
	Elac1	Nox4
	Tor3a	Prnp
	Cyp7a1	Itpr1
	Cyp4a3	Slc1a2
	Ctps1	Epm2a
	Mast3	Tmem86a
	Mus81	Serpina5
	Arg1	Slc26a8
	Rn45s	Slc20a1

Pcdha10	Hsph1
Pcdha4	Ddhd1
Ccl4	Helz2
Ubd	Ssx2ip
Kcnj15	Serhl2
Bank1	Ccnd2
Itgam	Bcl3
RGD1307603	Id2
Oasl	Tdrkh
Fcgr3a	Cldn2
Nkg7	Uhrf1
RT1-Ba	Mthfd2
Cd180	Srebf1
Irgm	Scrn1
Gpsm3	Slc7a7
Olr1353	Coprs
St8sia6	Dipk2a
Ifi47	Fam89a
LOC100910973	Dab1
Cacna1d	Myh14
Sgk2	Hsp90aa1
Ciita	Camkk2
Mx1	Plk2
Olr1584	Akap5
Xcl1	A1bg
Crym	Spats2l
Chka	Slc45a3
Meiob	Tbxas1
Arhgap9	Car2
RT1-Db1	Dnajc24
Ly86	Pmepa1
Lck	Ccdc146
Popdc2	Ptx3
Cd8a	S1pr1
Igtp	Banp
Il15	Ldhb
RT1-S2	Hbb
Prtg	Khdrbs3
Tap1	Kcnj8
Gna15	Nectin1
Rac2	Mest
Ptprc	Asrgl1
Ccl3	Ckap4
Fcmr	Fahd1
Cd74	Cavin3
Hsd3b3	Hebp2
Hsd3b1	Gna14
Abcg8	Mid1ip1
Stat1	Mcpt2
Themis2	Car7
Nfe2	Kank2
RGD1561157	Tax1bp3
Klrk1	Lox
Psmb9	Plxna2
Rnasel	Tubb4b
Psmb8	Dusp11
Upp2	Slc22a15

Fut1	Irs2
Cox11	Epha2
Leap2	Tmem176b
Slc16a6	Tdo2
Onecut1	RGD1566134
Otub2	Map3k5
Phlda3	Map1lc3b
Ogfod1	RGD1564804
Slc3a2	Ubald1
Nrbf2	Rps9
Aph1b	Hpgd
Gdf15	Insig2
Pdxk	Cd34
Slc7a5	LOC100912041
Hcn3	Myo7b
LOC500035	RT1-M2
LOC100365921	Eci1
Ndc1	Chid1
Nnmt	Rsad1
Coq7	Klf11
Amn1	Sult1a1
Bco1	Pck1
Fbxo44	Syde2
Tgfb2	Per3
Sptan1	Rapgef4
Paqr4	Nlrp12
Slc30a3	Ass1
Gtpbp3	Engase
Klhl25	LOC100365773
Dpep1	Ctse
Ceacam20	Fem1a
Mrm3	Il6r
Rsl1d1l1	Myo19
Nupr1	LOC102549726
Pcsk4	Acaa2
Pdhb	Dqx1
Kif22	Piga
Cntf	Ctif
Rn28s	Hprt1
Slc29a2	Nrg4
Pigb	Pex11a
Atl2	C17h6orf52
Snx8	Mettl15
Tjp3	Ahr
Purg	Tbx20
Synj2	Brap
Nek2	Fbxo31
Sez6	Slc37a4
	Hopx
	Fam126b
	Slc4a4
	Ppargc1b
	LOC100909675
	Abhd15
	Zfat
	Acer2
	Sycp3

Bcl6	Nedd9
Zbp1	Tspo
RT1-Da	Atp2b2
LOC691141	Dcp2
Npas2	Scd
Adamts7	Ddit4
Fcrla	Tubb6
Cmpk2	
Fgd2	
Itpripl1	
Car1	
Prkcb	
Tmem229b	
Il33	
Cd5l	
LOC688090	
RT1-M3-1	
RT1-DMb	
Oasl2	
Uchl3	
Plpp4	
Cd53	
Limd2	
Csgalnact1	
Emb	
Efna5	
Slc35c2	
Layn	
Gzma	
Plaat3	
Oas2	
Triap1	
Marco	
Pim1	
RT1-CE12	
Fads2	
RT1-Bb	
Pdgfc	
Arrdc3	
Ccl21	
Neurl3	
Vopp1	
Fcgr1a	
RT1-CE1	
Cplx2	
Prkcd	
Apoa2	
Aplp1	
Map4k1	
Dusp6	
Ccl6	
Tlr2	
Spry4	
Slamf7	
Arhgap8	
Parp9	
Parp14	

Ccpg1os
Zrsr1
Riox1
Exoc7
Pla2g15
Dhdds
LOC100360095
Tuft1
Cpt2
Ttll13
Fhl2
Tp53i13
Cyp4a1
Cyp2c11
Mat1a
Pex6
Marchf1
Gar1
Tmem203
Klf15
Cd151
Triap1
Mt1
Hps4
Tfcp2
Lhx2
Xbp1
Cds1
Taf1d
Psmd10
Il1b
Arhgap11a
Pla1a
Gpd2
Klf9
Plin2
Nol8
Ptger1
Cyp1a2
Rxylt1
Acot4
Tp53inp1
Ciart
Clec4m
Tmem63c
Bbs2
C1qtnf5
Fastkd1
LOC690617
Bbs1
Cyp2j4
Nudt4
Lrrc20
Taf11
Ablim1
RGD1307603
Camta1

Foxa2
Rnf125
Sla
Tmem184c
Phlda1
Slpi
Tmem150b
Opn3
Pstpip1
Ptpre
Nr0b2
Tor4a
Cd300a
Bcl2l11
Epsti1
Crip1
Rgs18
RT1-N3
Acsm3
Ptgs1
Fgr
Tmem159
Lilrb3a
Lilrb3b
Upp1
Gpx7
Nr1i3
Rsad2
B9d1
Etv5
Fyb1
Fabp7
Sipa1
Cd274
Il2rg
Samhd1
LOC102547290
Ifit3
Plcxd2
Usp18
Ggta1
Cdc14a
Inmt
Cd7
Tank
Megf8
Serpinb9
Plvap
Cyth4
Loxl4
Acnat2
Irf7
Hcst
Cmklr1
Adgre1
Crip2
Slamf8

Lepr
Lilrb3a
Lilrb3b
Enc1
Ftcd
Dao

Tfec
Anxa3
Hpgds
Alox5ap
Slc46a3
Alas2
Rad51d
Rap2b
Hsd3b5
Plscr2
Lgals5
Hcls1
Nox4
Fli1
H19
Tmem140
Spon2
Cited2
Tyrobp
Tfpi2
Pkdcc
Clec4a1
Mturn
Tmcc3
Csf1r
Parvg
C1s
Aif1
Serpinb6b
Il18
Olr35
Tlr7
Fxyd5
Gnpda2
Adh1
Lrrc8c
Oaf
Abhd5
Tsc22d4
Sgk1
Unc119
Slc15a3
Aacs
RT1-T24-3
Steap3
Cmtr2
Slc22a4
Obp3
Ncf4
Skap2
Exo5
Adgre4
Tifa
Itgal
Tppp
Zfp608
Carhsp1

Ccl27
Cd38
Prf1
Nlrc4
Bend7
Plcg2
Spred2
Tm7sf2
Rhpn2
Fzd5
Akr1c1
Dram1
Casp1
Abi3
Acsl3
Napsa
Bcl2a1
Tnfrsf1b
Sccpdh
Gls
Nfil3
Rfxap
St8sia4
Hao2
