# Supplementary material for: Similarity and dissimilarity in alterations of the gene expression profile associated with inhalational anesthesia between sevoflurane and desflurane
Source: PLoS One. 2024 Mar 28;19(3):e0298264. doi: 10.1371/journal.pone.0298264 (PMC10977671; doi:10.1371/journal.pone.0298264)
Supplement: S2 Table — (PDF) [file pone.0298264.s006.pdf]

Table S2

Gene lists denoted as leading-edge genes by GSEA

A

Leading edge genes for the term of "Drug Metabolism Cytochrome P450" identified as positively regulated event by desflurane treatment

ALDH3A1	ALDH3B1	FMO2	FMO3	CYP3A43	CYP3A7	GSTM5	CYP2B6	CYP2C18	CYP1A2
FMO5	FMO1	GSTP1	FMO4	GSTM3	CYP2E1	ADH4	UGT1A10	ALDH1A3	

B

Leading edge genes for the term of "Adaptive Immune Response" identified as negatively regulated event by sevoflurane treatment

TEC	C4A	MBL2	SEMA4A	ADA	STX7	CD209	C4BPA	CD226	C4B
CD28	RAG1	PIK3CG	IL1RL1	GZMM	CTSH	PKN1	TNFSF13B	CTSC	RNF8
CEACAM1	KCNJ8	PYCARD	ADAM17	IL6ST	SLA2	HLA-DPA1	FGL1	LAG3	FADD
TREX1	ORAI1	IRF4	TNF	INPP5D	IL12A	HLA-F	C1QA	P2RX7	CLEC10A
HLA-E	APLF	CXCL13	TGFB1	HLA-B	C9	PRDM1	WAS	TP53BP1	RIPK2
C1QB	LEF1	KLHL6	C1QC	TRAF3IP2	B2M	C1RL	HLA-G	MCOLN2	TLR4
STAT6	JAK2	TAP2	SAMSN1	IL23A	SLAMF6	RAP1GAP	CD84	NECTIN2	HLA-DMA
SYK	C1R	HAVCR2	SASH3	CSF2RB	RIPK3	DUSP10	MYO1G	BCL3	UNC93B1
IL18BP	KLRD1	CCL19	NLRP3	CR2	BACH2	CLEC4A	LY9	PLA2G4A	NCKAP1L
PIK3CD	CTSS	NLRP10	SLC11A1	IL2RB	ARID5A	C6	IL1B	IL21R	FCER2
FCER1G	RNF19B	TNFRSF1B	PTPRC	FZD5	PRF1	IL18	C1S	ARG2	LAT2
ADGRE1	IRF7	CD7	ADCY7	CD274	SIPA1	HLA-DOA	RSAD2	LILRB3	TREM1
CD247	RNF125	CD55	SLAMF7	PRKCD	FCGR1A	HLA-DQB1	CD6	GAPT	HLA-DMB
MICB	FUT7	SIT1	IL33	CD27	PRKCB	LAX1	HLA-DRA	ITK	CLEC4D
KLRC1	BCL6	KLRK1	CD74	SPN	CD8B	NOD2	TAP1	CD8A	HLA-DRB1
CD3E	CD3D	XCL1	ASCL2	SOCS3	HLA-DQA1	FCGR3A	HLA-DOB	CAMK4	IRF1
RAET1E	BATF	TBX21	SKAP1	IL27RA	BTLA	TNFRSF14	CD79B	FOXP3	IL18R1
IL10	SH2D1A	LTA	PRR7	VTCN1	SIGLEC10	LAIR1	CD40LG	CRTAM	SCART1
IFNG	PDCD1	IL9R	EBI3	PDCD1LG2	IL12B	TRAT1			

C

Leading edge genes for the term of "Cytokine Cytokin Receptor Interaction" identified as negatively regulated event by sevoflurane treatment

TNF*	FLT4	IL12A	CCR5	KITLG	KIT	CCL24	VEGFB	CXCL13	TGFB1
TNFSF10	LTB	EDA	PDGFRB	CXCL11	CSF1	IL1A	IL10RA	IL23A	CSF3R
OSM	CCR1	CSF2RB	CCL2	CCL19	CCR10	CXCL9	IL3RA	IFNLR1	IL2RB
IL1B	IL21R	TNFRSF1B	INHBA	CCL27	CCL22	IL18	CSF1R	FASLG	IL2RG
CCL23	CCL21	PDGFC	RELT	IL7	CD27	IL12RB2	INHBB	CCL3	IL22RA1
IL15	EPOR	XCL1	EDAR	CCR7	XCR1	CCL4	AMH	CXCL10	CXCL1
NGFR	TNFRSF14	PPBP	IL17B	BMPR1B	IL18R1	IL10	CCR9	TNFSF15	LTA
TNFRSF8	IL1R2	IL5RA	CD40LG	CSF2	CCL17	CCL25	IFNG	TNFSF11	IL9R
TNFSF9	TNFRSF18	CCL1	IL12B	CCR3					

*** genes common to D were indicated by red letters**

D

Leading edge genes for the term of "Cytokine Cytokin Receptor Interaction" identified as negatively regulated event by desflurane treatment

AMHR2	TGFBR1	CTF1	PF4	EDA*	IL17RA	IL12RB2	CXCL12	CCL26	CXCL13
TNFRSF21	XCL1	CCL27	IL2RG	IL23A	PDGFA	PDGFB	CCL21	CXCL16	FAS
CXCR5	TNFRSF25	INHBC	XCR1	PDGFRB	TNFRSF11B	CCL3	CXCR6	CCR1	TNFRSF1B
TNFRSF4	CCL28	CXCL10	PRLR	CNTF	CXCR3	TNFSF13	VEGFD	IL12RB1	IL2RA
IL12B	EDAR	TNF	CXCR2	TNFRSF17	IL10	IL17B	IL7	CCL2	INHBA
CCL7	CCR9	CCR6	PPBP	CSF2	IL1R2	LTA	OSM	IFNA4	IL20RB
TNFSF15	IL24	IL11	IL9R	CCR10	TNFSF8	TNFRSF18	IL12A		

*** genes common to C were indicated by red letters**
