# Supplementary material for: Similarity and dissimilarity in alterations of the gene expression profile associated with inhalational anesthesia between sevoflurane and desflurane
Source: PLoS One. 2024 Mar 28;19(3):e0298264. doi: 10.1371/journal.pone.0298264 (PMC10977671; doi:10.1371/journal.pone.0298264)
Supplement: Attachment — Submitted filename: Response to reviewers (PONE-D-23-16299).docx [file pone.0298264.s007.docx]

Response to Reviewers

**Reviewer #1:** 

**First comment from reviewer #1**

In order to be able to make the statement that circadian rhythm genes are differentially expressed after anesthesia with either sevoflurane or desflurane the authors need to control for circadian timing of the tissue harvesting. The authors do not share any information on harvesting time of the control group samples or the anesthetized samples.
From the methods the impression is made that rats were anesthetized at ZTx for 6hr and sacrificed at ZTx+6, whereas the control group was briefly induced at ZTx and immediately sacrificed. If this is the case, the harvested tissue of the anesthetized rats would be at a different circadian phase, and therefore would have different circadian rhythm gene expression than the controls. However this would be the effect of harvesting time rather than that of the anesthetic used.

As this reviewer pointed out, we had harvested livers from control and experimental rats at 9 am and 3 pm, respectively. Therefore, we conducted the same experiments in which all livers, including those in the control group, were harvested at 3 pm. Then, we compared the expression levels of circadian genes by quantitative PCR using RNAs prepared from the newly harvested liver samples. We found minimal differences in the expression levels between liver RNAs from control and anesthetized rats, indicating that the marked activation of circadian rhythm-related genes we observed previously were not due to inhalation anesthesia but rather the difference in their circadian rhythm phase. Because of this finding, we repeated the analyses using data from the newly conducted RNA-sequencing to revise our manuscript.

**Second comment from reviewer #1**

In addition, this raises the question whether any of the expression changes that the authors see is caused by other oscillating factors, like corticosterone levels for instance. We know that drug metabolism changes throughout the 24-h period, so the difference in gene expression for drug metabolism might also be attributed to the harvesting time. It doesn't seem like the authors have controlled for, or addressed this possible confounder.

As this reviewer stated, we found that not only canonical circadian genes but also a substantial number of genes including those for xenobiotic metabolism such as *Alas1* and *Por* were substantially affected by the timing of tissue harvesting. However, unlike circadian genes, the anesthetic procedure-dependent activation of these genes was still evident in the newly conducted analyses, albeit to a lower level than before.

**Third comment from reviewer #1**

Furthermore, the authors do not state the number of animals used for any of their experiments, and this makes it impossible to assess whether the study is well powered or not.

Although N=1 for the RNA-seq data, quantitative PCR used to validate the RNA-seq data were conducted with N=3. We described this explicitly in Methods section in the revised text (page 6 lines 16 to 18 for quantitative PCR and page 7 lines 3 to 5 for RNA-seq data).

**Fourth comment from reviewer #1**

As a minor issue with the availability of sequencing data, the reviewers were not provided a token to access the data, and GEO would not provide one but they said that the publisher would provide one. I didn't follow that up with the journal however.

We must apologize for this issue. We did not know that we had to create and provide a token so that any reviewers can access the private data. We have rectified this and provide a token (snqnkssarfwjjip) for our RNA sequence data (GSE244436). Once again, we would like to apologize any inconvenience that this reviewer encountered because of this issue.

**Reviewer #2:**

**First comment from reviewer #2**
In general, the result section needs to provide more details. Specially, there is no information about the computational and bioinformatic pipelines for RNA-seq data process and DEG identification. Please, provide them in the method section.

According to this reviewer’s comment, we provided the details of pipelines for the RNA-seq data process and DEG identification in the Methods section (page 7 line 1 to page 8 line 4). We also added information about the timing of tissue harvesting in the Methods section (page 5 line 18 to page 6 line 2)

**Second comment from reviewer #2**

The RNA sequencing data should not contain small RNA signals because these are depleted during size selection step to remove adaptors. However, there are small RNA signals at Table S1 (e.g. miRNA genes) and Figure 3S (e.g. MIRNA_MEDIATED_GENE_SILENCING_BY_INHIBITION_OF_TRANSLATION). Please, count aligned reads without small RNA genes (<= 200bp) and recalculate TPM values for GO and GSEA analyses. For the analyses, use liver expressed background gene sets that do not include the small RNA genes.

Because we did not know how to delete the data of the small RNA genes (≤200 bp), we removed all non-coding RNAs from the list including longer RNAs (>200 bp) from the list using BioMart web tool (<https://asia.ensembl.org/info/data/biomart/index.html>) as described on page 7 lines 14 to 15 and conducted GO and GSEA analyses in response to this reviewer’s comment. We hope that these procedures are satisfactory for this reviewer.

**Third comment from reviewer #2**

In the method section, zeitgeber times are not very clear to collect 0-hour and 6-hour treatment samples. If there is 6-hour gap between the samples, there is additional factor of time that alters the expression of gene sets related to daily circadian rhythms including sevoflurane and desflurane treatments. The phase of the circadian rhythm during treatment with either anesthetic could very likely be the driver of the differential gene expression if there is 6-hour gap between the samples. To reach the same conclusion, the author needs to subtract the rhythmic gene expression changes.

As this reviewer and other reviewers pointed out, there was a 6-hour gap in liver harvesting time between the control and experimental rats. Therefore, we conducted the same experiments in which all livers, including those of the control group, were harvested at the same time, at 3 pm. Then, we compared the expression levels of circadian genes by quantitative PCR using RNAs prepared from newly harvested liver samples and found only minimal differences in their expression levels between liver RNAs from anesthetized rats and control rats. This indicated that the marked activation of circadian rhythm-related genes we observed previously was not due to inhalation anesthesia but rather to the difference in their circadian rhythm phase. Because of this finding, we repeated the analyses using data from newly conducted RNA-sequencing to revise our manuscript.

**Fourth comment from reviewer #2**

Main figures need higher resolution since it is very hard to read the figures.

In the revised manuscript, we provided figures with a high resolution (600 dot per inch). We hope that the resolution of the newly provided files is satisfactory.

**Fifth comment from reviewer #2**

For qPCR experiments, provide if t-tests were one- or two-tailed. Also, note how many samples are used.

A two-tailed *t*-test (N=3) was conducted for all experiments. We described this in Methods section (page 8 lines 15 to 17) as well as in legends of Figure 4 (page 34 lines 4 to 6) in the revised text.

**Sixth comment from reviewer #2**

For Fig. 3, provide more explanation in the figure legend to understand the figure.

We have provided information related to the number of genes included in each gene set and how many genes were activated or repressed. We hope that our response is satisfactory.

**Seventh comment from reviewer #2**

For Fig. S2B and Fig. 2D, heatmaps showed similar reduced expression at the GO terms. It is better to include plots that show expression correlations with R2 and p-values.

We provided these data in Fig. S2 and S3 in the revised manuscript. These regression analyses revealed that neither xenobiotic metabolism-related genes nor immune response-related genes had a noticeable correlation related to variations in their expression levels between the sevoflurane and desflurane groups. We described about these results in the text (page 11 lines 1 to 7, pages 13 lines 5 to 11 and page 18 lines 10 to 17).

**Eighth comment from reviewer #2**

For the Venn diagrams at fig 1C and 2C, provide hypergeometric p-values to show how significantly the gene sets are overlapped.

As suggested, we provided these data in Fig. 1A and Fig. 2A in the revised manuscript.

**Reviewer #3:**

**First comment from reviewer #3**

This paper concerns differences in gene expression in response to two different inhalation anaesthetic agents (sevoflurane and desflurane). I must admit to being a little confused about the overall point of the study and the results presented. While differences in a number of different gene expression profiles are presented (particularly for genes involved in the molecular mechanics of the circadian clock and in drug metabolism) there were no differences in the expression changes between the two agents. The authors do report a downregulation in the expression of cholersterol biosynthesis genes in response to sevoflurane but I am unsure of the potential clinical significance of this finding. One significant omission from the manuscript was any description of the timing of the administration of the two agents. Particularly with respect to the analysis of circadian gene expression profiles this is very important. Time of day will have a profound influence on the level of expression of clock genes (and also potentially on the influence of the anaesthetic agents on the expression of clock genes).

As this reviewer and other reviewers pointed out, there was a 6-hour gap in liver harvesting time between the control and experimental rats. Therefore, we conducted the same experiments in which all livers, including those of the control group, were harvested at the same time, at 3 pm. Then, we compared the expression levels of circadian genes by quantitative PCR using RNAs prepared from newly harvested liver samples and found only minimal differences in their expression levels between liver RNAs from anesthetized rats and control rats. This indicated that the marked activation of circadian rhythm-related genes we observed previously was not due to inhalation anesthesia but rather to the difference in their circadian rhythm phase. Because of this finding, we repeated the analyses using data from newly conducted RNA-sequencing.

GO analyses using data from the newly conducted RNA-sequencing revealed that terms related to circadian rhythm were no longer significant terms with sevoflurane-treated sample, although the term “circadian regulation of gene expression (GO:0032922)” was identified with desflurane-treated sample in the 12^th^ position from the top. Unexpectedly, the GO term “cholesterol biosynthetic process (0006695)” was not identified with samples from rats treated with sevoflurane, indicating that a 6-hour gap in tissue harvesting not only affected canonical circadian genes in their expression levels but also affected many other genes whose expressions oscillate between day and night. These findings indicate that adjusting the timing of tissue harvesting within the day are critical when comparing control and experimental animals.

We would like to thank this reviewer and other reviewers because we would not have conducted the RNA-sequencing analyses again without these invaluable comments.

**Second comment from reviewer #3**

I did not get a clear idea of the point of the manuscript. I think the manuscript would benefit from a substantial re-work in order to make it clear why the authors would expect changes in the gene expression profiles they have seen and what the scientific and clinical relevance of this changes might be.
We have intensively edited the text, especially in the Abstract (page 2 lines 2 to 9) and Introduction (page 4 lines 3 to 9) in response to this reviewer’s comment. We edited the text with a focus on the comparison of the effects of sevoflurane and desflurane on the expressions of genes related to xenobiotic metabolism and immune response, because no comprehensive comparison has been reported to date. We hope the newly edited text is satisfactory.

**Third comment from reviewer #3**

GA is not sedation.

To avoid confusion, we eliminated this expression from the text.

**Fourth comment from reviewer #3**

Isoflurane is the most commonly used inhalational agent in the world not sevoflurane

As suggested, we changed the expression from “the most---” to “one of the most---” (first line in the abstract and second sentence in the Introduction section).
