# Supplementary material for: Similarity and dissimilarity in alterations of the gene expression profile associated with inhalational anesthesia between sevoflurane and desflurane
Source: PLoS One. 2024 Mar 28;19(3):e0298264. doi: 10.1371/journal.pone.0298264 (PMC10977671; doi:10.1371/journal.pone.0298264)
Supplement: Attachment — Submitted filename: Nogi et al. response to reviewers.docx [file pone.0298264.s008.docx]

Response to Reviewers

**Reviewer #1:** 

**First comment from reviewer #1**

The way it is presented is very difficult to follow. Below I have highlighted some segments that are examples.
example 1: this following segment is one sentence.
"In accordance with these data, regression analysis demonstrated no apparent correlation in alterations of the expressions of these genes between sevoflurane and desflurane treatments in case that data of Cyp2b1 expression were removed as an outlier in the gene set (S2A Fig, lower panel), although an analysis of all genes shown in Figure 1D suggests a high correlation (R2=0.9371) because of the marked activation of Cyp2b1 gene expression by both anesthetic agents (S2A Fig, upper panel).

We agree the English in this section was extremely difficult to follow as it was difficult to explain the data explicitly, because the regression analyses conducted according to a comment from another reviewer with a gene set shown in Figure 1D apparently contradicted the result obtained from the heatmap visualization. However, in the revised manuscript, this section of the text was edited extensively with the aid of professional English editing service so that it can be understood more easily (page 10 lines 1 to 8). We hope that newly edited text is satisfactory.

example 2: this following segment is one sentence.
"Although no GO term related to immunological response was obtained in the analyses of genes downregulated by desflurane, we inspected the gene expression levels in the livers of rats subjected to inhalational anesthesia with desflurane as to genes that were contributed to the identification of the GO term, “defense response to virus” (GO:0051607) as sevoflurane- specific terms because of their downregulation more than 2-fold by sevoflurane treatment."

Again, we agree this section was extremely difficult to follow. We did not use an entire gene set constituting the GO term “defense response to virus”, but rather selected genes with expression levels reduced more than 2-fold by sevoflurane treatment from the gene set. Accordingly, we have rewritten the text in the revised manuscript with the aid of a professional editing service (page 12 lines 10 to 16) and we hope this is now easier to understand.

Example 3: this following segment is one sentence.
Regression analyses revealed that, although there was a weak correlation between genes that contributed to the identification of the GO term, “response to bacterium” (GO:0009617) as a sevoflurane treatment-specific term (R2=0.2605) (S3B Fig), no appreciable statistical significance was apparent with other gene sets (genes downregulated more than 2-fold by sevoflurane treatment among genes comprising the terms of GO:0051607 and GO:0006955) (S2B Fig and S3A Fig).

We also edited this portion extensively so that it is easier to understand (page 13 lines 7 to 15). We hope that newly edited text is satisfactory for this reviewer.

**Second comment from reviewer #1**

The manuscript is overwhelming with these kind of sentencing constructs.

In the revised manuscript, we tried to avoid making overstatements. In particular, we removed the following sentences from the Discussion section, because we felt that they were too exaggerated and could be removed without affecting the scientific content.

“This finding might alter the current concept that one of major side-effects of general anesthesia with a volatile anesthetic agent is immunosuppression.”

**Reviewer #2:**

**Comment from reviewer #2**
The revised manuscript has appropriately addressed most of my comments. However, there is one aspect where the authors need to make a correction in their approach.

To delete the data of the small RNA genes (≤200 bp), removing all non-coding RNAs is not the optimal approach. While it effectively eliminates most small RNAs, it could also lead to the depletion of all long non-coding RNAs, remaining some small RNAs originate from protein-coding genes.
According to this reviewer’s comment, we removed the small RNA genes (≤200 bp) from the gene list instead of removing all the non-coding genes, and re-conducted all the analyses shown in Figures 1-3 and all the supplementary figures and tables.
